# Coevolution of Metabolic Pathways in Blattodea and Their *Blattabacterium* Endosymbionts, and Comparisons with Other Insect-Bacteria Symbioses

**DOI:** 10.1128/spectrum.02779-22

**Published:** 2022-09-12

**Authors:** Yukihiro Kinjo, Thomas Bourguignon, Yuichi Hongoh, Nathan Lo, Gaku Tokuda, Moriya Ohkuma

**Affiliations:** a Okinawa Institute of Science and Technology Graduate University, Okinawa, Japan; b RIKEN Bioresource Research Centre, Tsukuba, Japan; c School of Life Science and Technology, Tokyo Institute of Technology, Tokyo, Japan; d Faculty of Tropical AgriSciences, Czech University of Life Sciences, Kamýcká,Prague, Czech Republic; e School of Life and Environmental Sciences, University of Sydney, Sydney, New South Wales, Australia; f Tropical Biosphere Research Center, University of the Ryukyus, Okinawa, Japan; University Of Thessaly

**Keywords:** *Blattabacterium*, Blattodea, co-evolution, co-metabolism, endosymbiosis, insects

## Abstract

Many insects harbor bacterial endosymbionts that supply essential nutrients and enable their hosts to thrive on a nutritionally unbalanced diet. Comparisons of the genomes of endosymbionts and their insect hosts have revealed multiple cases of mutually-dependent metabolic pathways that require enzymes encoded in 2 genomes. Complementation of metabolic reactions at the pathway level has been described for hosts feeding on unbalanced diets, such as plant sap. However, the level of collaboration between symbionts and hosts that feed on more variable diets is largely unknown. In this study, we investigated amino acid and vitamin/cofactor biosynthetic pathways in Blattodea, which comprises cockroaches and termites, and their obligate endosymbiont Blattabacterium cuenoti (hereafter *Blattabacterium*). In contrast to other obligate symbiotic systems, we found no clear evidence of “collaborative pathways” for amino acid biosynthesis in the genomes of these taxa, with the exception of collaborative arginine biosynthesis in 2 taxa, *Cryptocercus punctulatus* and *Mastotermes darwiniensis*. Nevertheless, we found that several gaps specific to *Blattabacterium* in the folate biosynthetic pathway are likely to be complemented by their host. Comparisons with other insects revealed that, with the exception of the arginine biosynthetic pathway, collaborative pathways for essential amino acids are only observed in phloem-sap feeders. These results suggest that the host diet is an important driving factor of metabolic pathway evolution in obligate symbiotic systems.

**IMPORTANCE** The long-term coevolution between insects and their obligate endosymbionts is accompanied by increasing levels of genome integration, sometimes to the point that metabolic pathways require enzymes encoded in two genomes, which we refer to as “collaborative pathways”. To date, collaborative pathways have only been reported from sap-feeding insects. Here, we examined metabolic interactions between cockroaches, a group of detritivorous insects, and their obligate endosymbiont, *Blattabacterium*, and only found evidence of collaborative pathways for arginine biosynthesis. The rarity of collaborative pathways in cockroaches and *Blattabacterium* contrasts with their prevalence in insect hosts feeding on phloem-sap. Our results suggest that host diet is a factor affecting metabolic integration in obligate symbiotic systems.

## INTRODUCTION

Many insects have established intimate relationships with bacterial symbionts that have helped them to exploit new niches. In many cases, intracellular symbionts provide nutrients to their insect host, while the host provides a stable environment favorable to symbiont growth (1, 2). These symbiotic relationships are often obligate, as the host and its symbionts depend on products encoded in the genome of their partners. Moreover, obligate symbioses are known to involve host-symbiont complementarity, whereby each partner relies on metabolic products and enzymes of the other to complete some of their metabolic pathways.

One example is branched-chain amino acid (BCAA) biosynthesis in aphids and their endosymbiotic Buchnera aphidicola (hereafter *Buchnera*). Aphids depend on *Buchnera* to supply many essential amino acids (EAAs) deficient in their phloem-sap diet (3, 4). However, the genome of *Buchnera* lacks the genes coding for branched-chain amino acid aminotransferase (*ilvE*), the enzyme involved in the last step of BCAA biosynthesis (5), which is present in the aphid genome (6). The genomes of both symbiotic partners are therefore complementary, sometimes at the level of genes within pathways, and together encode all genes involved in EAA biosynthesis (4, 7–9).

Such intimate metabolic interactions, which we term “collaborative pathways” (10), are not unique to aphids and have also been found in several other host-symbiont systems (e.g., 11, 12). Collaborative pathways typically evolve from hosts and symbionts endowed with complete functional pathways at the origin of symbiogenesis, but may depend on genes horizontally transferred from other bacteria to the host. In addition to the preexisting gene set of the symbiont pair, collaborative pathways require the evolution of unusual transport mechanisms for metabolites or enzymes between members of the pair. Collaborative pathways have been reported from obligate symbiotic systems that have a long history of coevolution (10, 13).

To date, the existence of collaborative pathways between hosts and symbionts has only been investigated in sap-feeding insects (13). Although a collaborative arginine biosynthetic pathway between carpenter ants and their obligate endosymbiont, *Blochmannia*, has been proposed based on the gene content of the symbiont genome (14), comparisons between the genomes of host insects with alternative diets and that of their symbionts have not yet been reported. A model for insect symbioses that do not involve sap-feeding taxa is that between Blattodea, which comprises cockroaches and termites (15), and their intracellular symbiont Blattabacterium cuenoti (hereafter *Blattabacterium*). Blattodea have evolved a wide range of feeding habits and are sometimes known as “garbage collectors,” recycling dead plants, dead animals, and excrement (16). Almost all cockroaches, as well as the most primitive termite *Mastotermes darwiniensis*, host *Blattabacterium* endosymbionts in specialized cells (bacteriocytes) located in their fat body. *Blattabacterium* appears to have been lost in the lineage leading to all termites other than *M. darwiniensis*, as it has not been found in these taxa. The association between Blattodea and *Blattabacterium* is ancient and was established >200 million years ago (17–19), which is comparable with the time frame of other symbiotic systems found in insects (e.g.160-280 MYA in aphids-*Buchnera*, [20]). Physiological experiments and genomic analyses have suggested that *Blattabacterium* is involved in nitrogen recycling and essential amino acid biosynthesis (21–23). Unlike sap feeders, cockroaches have adapted to various diets, and many species are known to engage in coprophagy (16). Their diet is therefore more nutritionally balanced than plant sap. However, because their food sources are often transient (16), *Blattabacterium* is likely to contribute to host fitness through nitrogen recycling. Thus, metabolic collaborations between the two genomes are expected to be different from that of sap-feeding hosts and their symbionts.

The evolution of metabolic pathways in *Blattabacterium* is largely unknown. In contrast to the endosymbionts belonging to the phylum *Pseudomonadata* (previously *Proteobacteria*), to which Escherichia coli belongs, the enzyme repertoire of the order *Flavobacteriales* (phylum *Bacteroidota*), to which *Blattabacterium* belongs, is not well-characterized. Unknown enzymes and pathways are still frequent in lineages that do not include model organisms, affecting prokaryote genome annotation (24). Indeed, many bacteria have genomes annotated with incomplete amino acid biosynthetic pathways and are incorrectly assumed to be heterotrophic while they can be cultured without the given amino acids (25). For this reason, it is unclear whether the apparent gaps present within the pathways of *Blattabacterium* are genuine gaps, representing gene losses during the evolution of *Blattabacterium* and complemented by their host Blattodea, or whether these genes are absent from the genomes of *Blattabacterium* and their free-living relatives because of the existence of yet-uncharacterized genes replacing the functions. Genes absent in all *Blattabacterium* genomes as well as the genomes of their free-living relatives are indicative of the presence of uncharacterized genes completing the pathways and are not expected to be compensated by the host genome.

In this study, we conducted a careful reconstruction of the metabolic pathways involved in amino acid and vitamin/cofactor biosynthesis of *Blattabacterium* and their host Blattodea. We investigated the genomic evidence of metabolic collaboration between host insects and *Blattabacterium*. To this end, we reconstructed the metabolic pathways of seven representative genomes of *Blattabacterium* and compared them to the metabolic pathways of other *Flavobacteriales* bacteria. This approach allowed us to distinguish genes that were lost in *Blattabacterium* and are complemented by their insect host from genes commonly absent in *Flavobacteriales* (whose inferred function is carried out by the products of yet-uncharacterized genes or pathways). We then inferred the metabolic pathways of Blattodea using the genome and transcriptome of 2 Blattodean species harboring *Blattabacterium* (*Periplaneta americana* and *Cryptocercus punctulatus*, respectively) and the genome of 1 Blattodean species devoid of *Blattabacterium* (*Zootermopsis nevadensis*). Using this approach, we obtained robust inference of the pathways common across Blattodea. We compared the inferred metabolic pathways of Blattodea and *Blattabacterium* and identified potential collaborative pathways. Finally, we compared the profile of collaborative pathways in *Blattabacterium* with those of other host-symbiont systems with variable diets to see how diet affects the profile of collaborative pathways.

## RESULTS

### Evolution of metabolic pathways in *Blattabacterium*.

**FIG 1:**
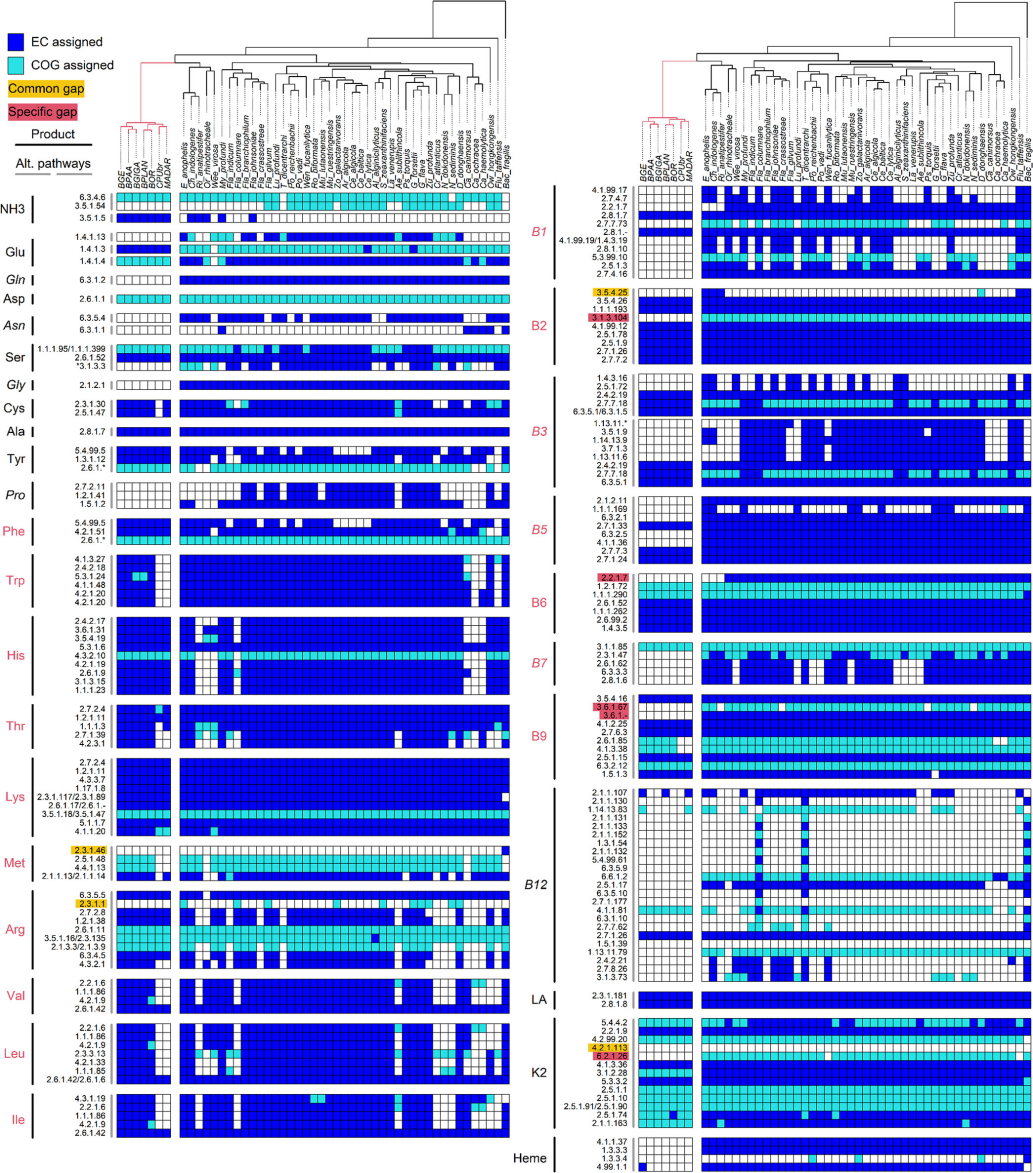
Metabolic capabilities of *Flavobacteriales* genomes. Presence/absence of the enzymes involved in amino acid and vitamin/cofactor biosynthetic pathways for 42 free-living *Flavobacteriales* strains and seven *Blattabacterium* genomes. The branches in the phylogenetic tree leading to *Blattabacterium* strains are shown in red. Blue boxes, EC-number-assigned reactions; light blue boxes, COG-assigned reactions; empty boxes, missing genes. The black and gray vertical bars indicate the final products of the pathways, and alternative pathways that produce the same product, respectively. The final products that are not likely produced by *Blattabacterium* are shown in italics. Amino acids and vitamins essential for Blattodea hosts are shown in red. EC numbers assigned as gaps within pathways are highlighted with the following colors: yellow, gaps common across *Blattabacterium* and other *Flavobacteriales* strains (>10% genomes); red, gaps generally specific to *Blattabacterium*. *the final reaction in the serine biosynthetic pathway (EC 3.1.3.3) was not assigned as gap-specific to *Blattabacterium* (see main text). LA, lipoic acid.

**FIG 2:**
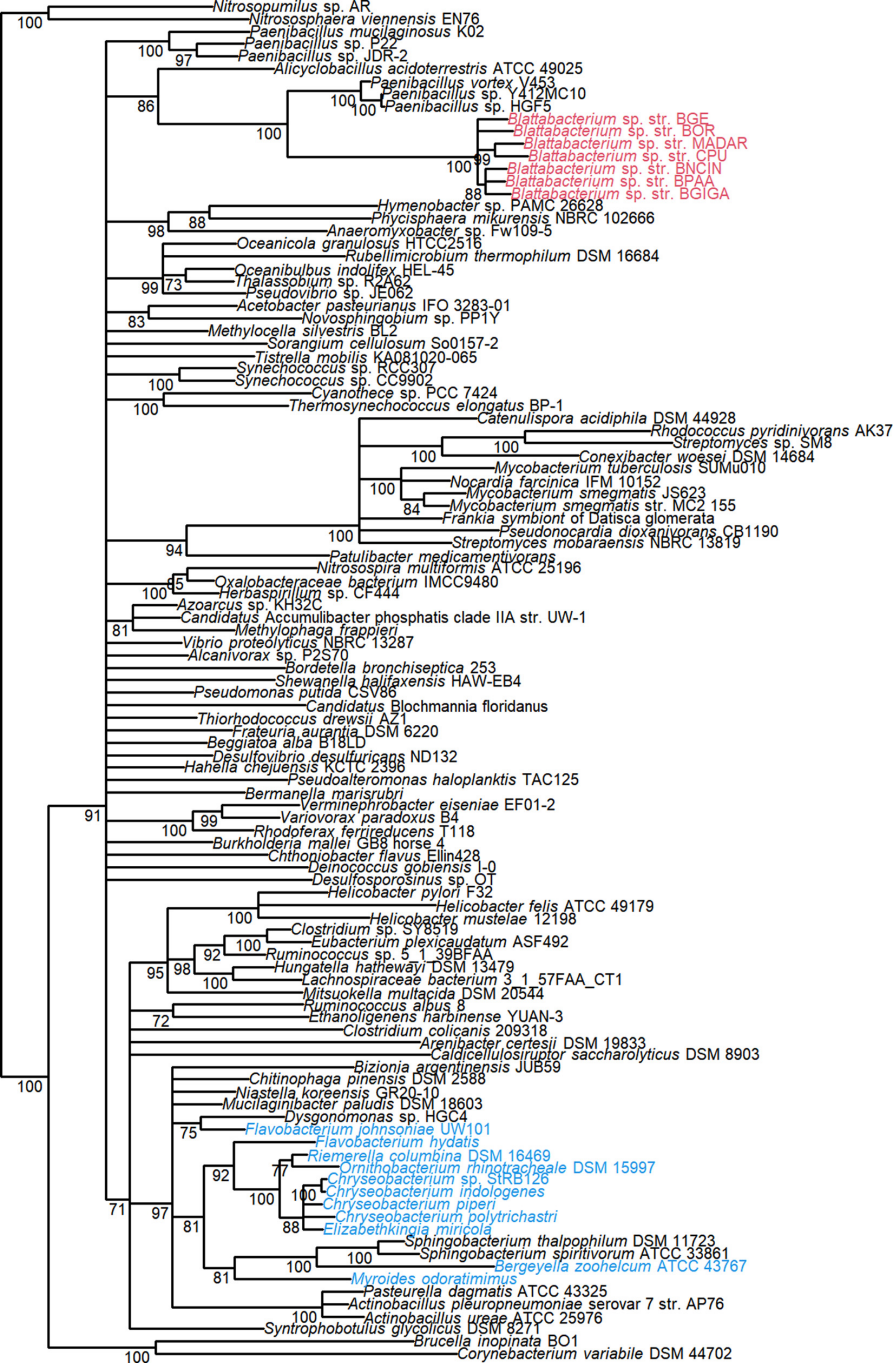
Phylogenetic analysis of the ureC gene in bacteria. Maximum likelihood phylogenetic tree of *ureC* gene reconstructed using RAxML with PROTCATLG amino acid substitution model. *Blattabacterium* and *Flavobacteriales* strains are colored in red and blue, respectively. Nodes with low (<70%) bootstrap support values were collapsed.

We analyzed the metabolic pathways present in 42 genomes of non-*Blattabacterium Flavobacteriales* bacteria and the genome of a representative of the genus *Bacteroides* of the Bacteroidales order, which we used as an outgroup (Table S1). We compared these with the metabolic pathways found in 7 *Blattabacterium* genomes. The presence/absence of each enzyme involved in amino acid and vitamin/cofactor biosynthesis is summarized for all genomes in Fig. 1. One unique characteristic of all *Blattabacterium* strains is the presence of urease (EC 3.5.1.5) in their genomes, a gene that is generally absent from the genomes of other strains of *Flavobacteriales*. Phylogenetic analysis of the urease large subunit (UreC) revealed that the sequences of *Blattabacterium* form a monophyletic cluster which is sister to sequences from members of the genus *Paenibacillus* (*Firmicutes*) (Fig. 2).

The biosynthetic pathways of four amino acids not essential in insects (hereafter non-EAAs), glutamine, asparagine, glycine, and proline, and two vitamins/cofactors, pantothenate (B5) and protoheme, were lost almost exclusively in *Blattabacterium* (Fig. 1). In contrast, the almost complete biosynthetic pathways of 10 amino acids essential in insects (including arginine (26); hereafter EAAs) were retained in all *Blattabacterium* strains, except in the strains hosted by *Cryptocercus punctulatus* (CPUbr) and *Mastotermes darwiniensis* (MADAR), which have lost several of these pathways (Fig. 1). At the within-pathway level, we identified several gaps (missing genes) in biosynthetic pathways. We defined gaps as missing enzymes (genes) in pathways otherwise complete. We recognized 2 categories of gaps: (i) “common gaps,” which are shared among *Blattabacterium* and over 70% of other *Flavobacteriales* genomes (EC numbers highlighted in yellow in Fig. 1); and (ii) “specific gaps,” which are generally specific to *Blattabacterium* (EC numbers highlighted in red in Fig. 1). Since common gaps are found in other strains of *Flavobacteriales*, regardless of their ecological habitats, and the other enzymes involved in the pathway are highly conserved, we assume gaps are complemented by alternative enzymes in the genomes of *Blattabacterium* and other *Flavobacteriales* rather than by metabolites obtained from the host (see Materials and Methods). On the contrary, specific gaps found only in *Blattabacterium* are likely to be complemented by host-derived metabolic intermediates, assuming that the host genome encodes genes complementing these gaps. Missing genes in the methionine, arginine, and riboflavin (B2; EC 3.5.4.25) biosynthetic pathways were assigned to common gaps. On the other hand, missing genes in the pyridoxine (B6), riboflavin (B2; EC 3.1.3.104), folate (B9), and menaquinone (K2; EC 6.2.1.26) biosynthetic pathways were assigned to the specific gap category. In addition, the enzyme involved in the final step of arginine biosynthesis, argininosuccinate lyase (ArgH; EC 4.3.2.1), and 2 enzymes involved in the synthesis of para-aminobenzoate (pabA and trpE; EC 2.6.1.85 and EC 4.1.3.38) from the folate biosynthetic pathway, were absent from the genomes of the *Blattabacterium* strains CPUbr and MADAR, and were therefore considered as specific gaps (Fig. 1). A gap in the serine biosynthetic pathway (EC 3.1.3.3) was not classified into any category as it is neither common across *Flavobacteriales* genomes nor specific to *Blattabacterium*.

### Metabolic relationships between *Blattabacterium* strains and their hosts.

**FIG 3:**
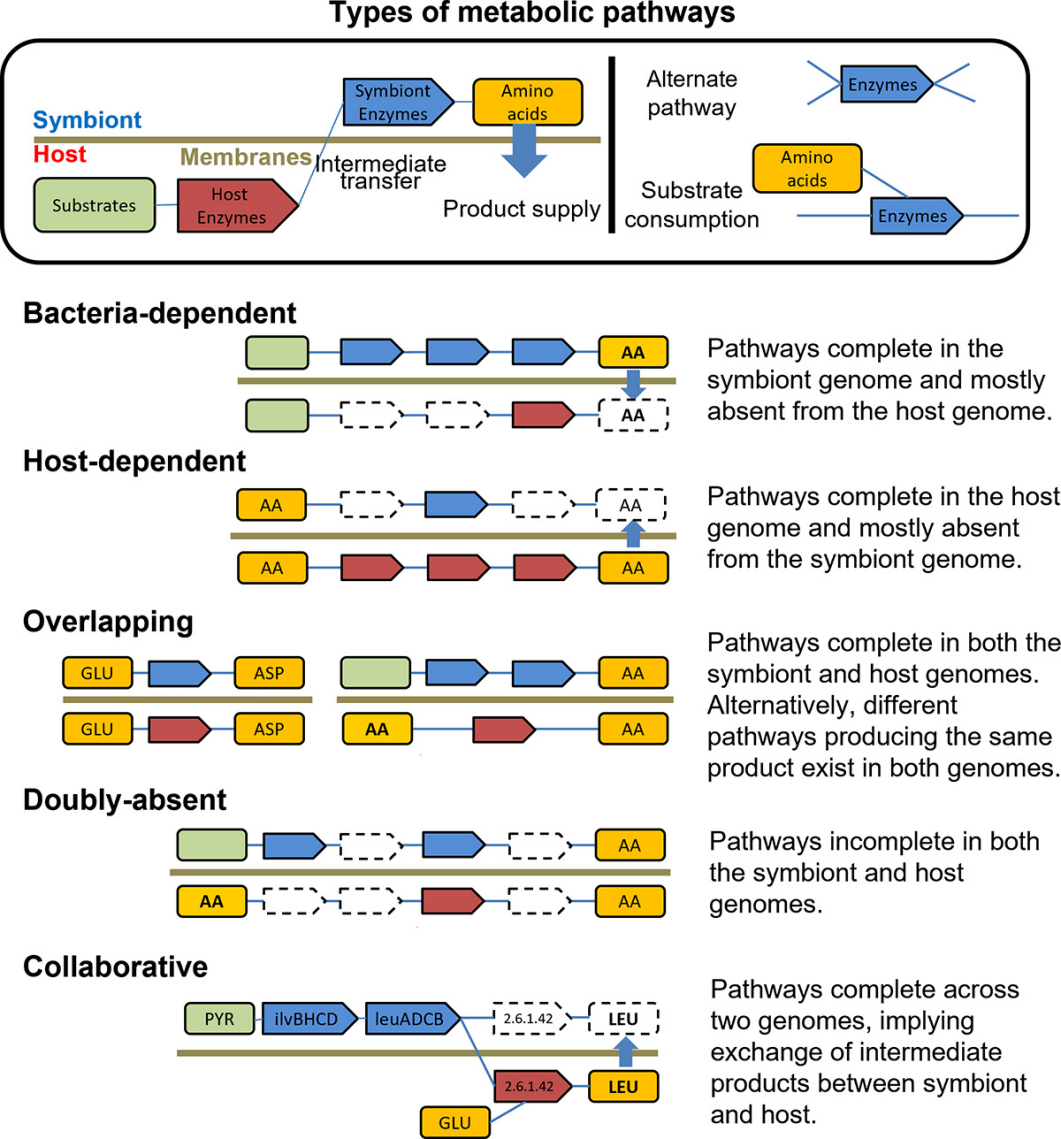
Types of metabolic interactions between *Blattabacterium* and their hosts. Boxes with connections represent biosynthetic pathways. Host enzymes and symbiont enzymes are represented by red and blue boxes, respectively. Blue arrows indicate products supplied to the symbiotic partner. Blue bars connecting box edges represent the routes of the pathways.

To investigate the molecular basis of *Blattabacterium*-host metabolic collaboration, we compared the reconstructed metabolic pathways of *Blattabacterium* to those of their hosts, with the assumption that gaps common across *Flavobacteriales* and present in *Blattabacterium* probably do not need to be compensated by their hosts. We inferred the common metabolic pathways of Blattodea representatives by reconstructing consensus pathways from the cockroaches *P. americana* and *C. punctulatus* and the termite *Z. nevadensis*. The metabolic relationships between *Blattabacterium* and their hosts were classified into five categories (Fig. 3): *Blattabacterium*-dependent, host-dependent, overlapping, doubly-absent, and collaborative pathways.

**FIG 4:**
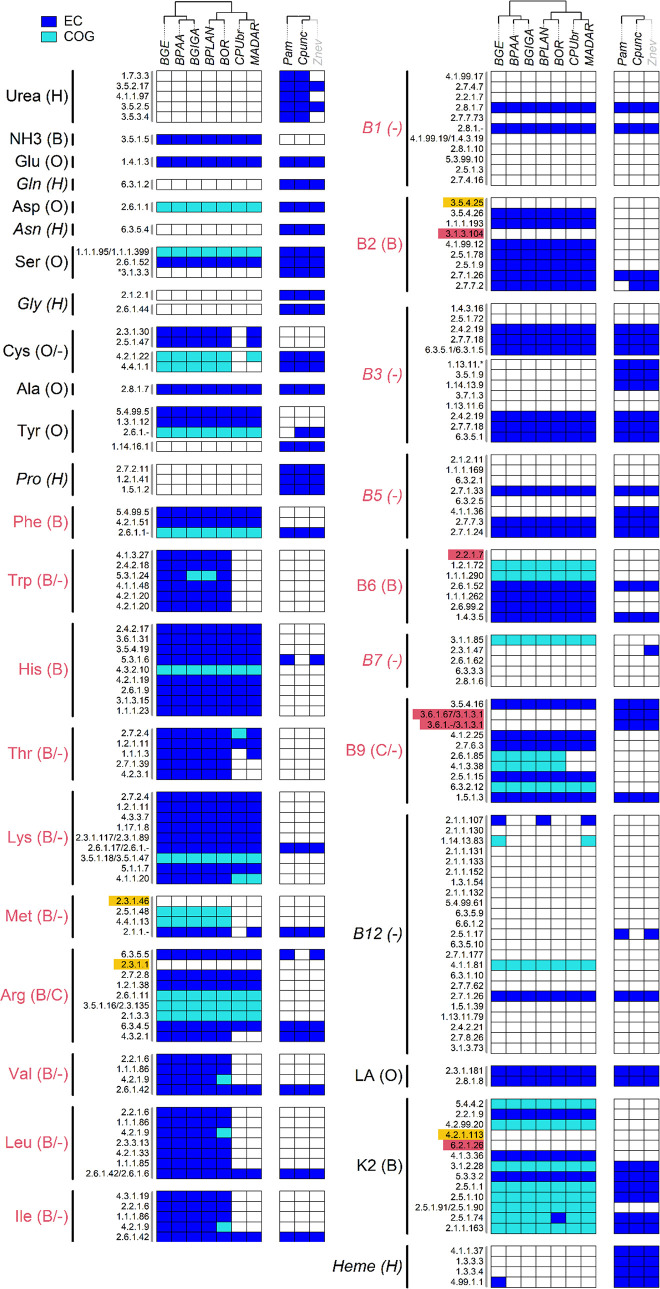
Pathway comparisons between *Blattabacterium* strains and their Blattodea host. Characters within parentheses next to the final products indicate: H, host-dependent; B, *Blattabacterium*-dependent; O, overlapping; -, doubly-absent; C, collaborative pathways.

The biosynthetic pathways of the 10 EAAs were all *Blattabacterium*-dependent except for CPUbr and MADAR. No horizontal gene transfers were detected for amino acid and cofactor biosynthetic pathways in *P. americana* and *Z. nevadensis* genomes. Conversely, non-EAA pathways were all host-dependent or overlapping. While the biosynthesis of glycine, asparagine, glutamine, and proline was categorized as host-dependent, the biosynthesis of serine, cysteine, alanine, tyrosine, glutamate, and aspartate was categorized as overlapping (Fig. 4). For the serine biosynthetic pathway, it is possible that the loss of SerB in *Blattabacterium* genomes is complemented by phosphoserine phosphatase (EC 3.1.3.3) derived from the host genome. However, SerB is also absent from the genomes of several *Flavobacteriales* strains and an alternative enzyme catalyzing the same reaction, HisB (EC 3.1.3.-) (27), is present in *Blattabacterium* genomes and may compensate the lack of SerB, as previously suggested (23). We therefore categorized the serine biosynthetic pathway as overlapping. The initial steps of the host-*Blattabacterium* metabolic pathway for nitrogen metabolism, the uricolytic pathways, are complete in the two cockroach transcriptomes investigated in this study. On the other hand, the lower termite *Z. nevadensis*, which does not harbor *Blattabacterium*, lacks three enzymes in the uricolytic pathway: urate oxidase (EC 1.7.3.3), 2-oxo-4-hydroxy-4-carboxy-5-ureidoimidazoline decarboxylase (EC 4.1.1.97), and allantoicase (EC 3.5.3.4) (Fig. 4).

*Blattabacterium* CPUbr and MADAR have undergone further genome reduction, being ~50kbp smaller than other strains (28, 29). The additional genome reduction in these strains is mostly due to the loss of many genes involved in EAA biosynthesis. It has been hypothesized that the loss of these genes occurred as a result of relaxed selection due to the provision of EAAs either by the gut symbiotic system established in the common ancestor of *C. punctulatus* and *M. darwiniensis* (28–31), and/or through social transfer of nutrients (proctodeal trophallaxis). One of these gene losses is present as a single gap in the arginine biosynthetic pathway, and consists of the loss of the gene encoding the enzyme involved in the final step of arginine biosynthesis, ArgH (EC 4.3.2.1). Although the products of the two *Blattabacterium* genes *purB* and *fumC* possibly replace the function of ArgH (30), the host genome also encodes arginosuccinate lyase (ASL: EC 4.3.2.1), an enzyme with a catalytic function similar to ArgH. The loss of ArgH has occurred in parallel in the ancestors of CPUbr and MADAR (29). A collaborative pathway involving the loss of ArgH by the endosymbiont *Carsonella* and complementation by a psyllid host gene was reported by Sloan et al. (2014), based on transcriptomic evidence. Therefore, we hypothesize that the arginine biosynthetic pathways of CPUbr and MADAR are collaborative (Fig. 4).

Among cofactor biosynthetic pathways, the riboflavin (B2), pyridoxine (B6), and menaquinone (K2) pathways were *Blattabacterium*-dependent (Fig. 4). The protoheme pathway was host-dependent, and the lipoate pathway was overlapping between *Blattabacterium* and their host (Fig. 4). The thiamine (B1), niacin (B3), pantothenate (B5), biotin (B7), and cobalamin (B12) biosynthetic pathways were doubly-absent (Fig. 4). Although we found several gaps in the riboflavin, pyridoxine, and folate pathways, we found no enzyme in the host genomes to compensate for these gaps, except for alkaline phosphatase (EC 3.1.3.1) of the folate pathway, suggesting that the pathways for pyridoxine and riboflavin biosynthesis use alternative enzymes, such as multifunctional enzymes, to catalyze these reactions. For the *Blattabacterium*-specific gap in the folate biosynthetic pathway, which comprises 2 reactions (EC 3.6.1.67 and EC 3.6.1.-), we identified a host enzyme, alkaline phosphatase (EC 3.1.3.1), which can bypass these reactions (32). Although we could not find any experimental evidence for this bypassed reaction, transcriptome-based evidence for this collaboration has been described in the symbiotic system composed of whiteflies and their secondary symbionts, *Hamiltonella* (33). We thus hypothesize that this pathway is a collaborative pathway in Blattodea, as is the case in whiteflies. *Blattabacterium* CPUbr and MADAR have additionally lost 2 enzymes in the folate biosynthetic pathway, PabA (EC 2.6.1.85) and TrpE (EC 4.1.3.38), which are involved in the *p*-aminobenzoate (PABA) synthesis. We found no enzymes in the host genomes compensating for the loss of the PABA branch. We therefore hypothesize that the folate biosynthetic pathway is doubly-absent in CPUbr and MADAR.

### Comparison with other host-symbiont partnerships.

**FIG 5:**
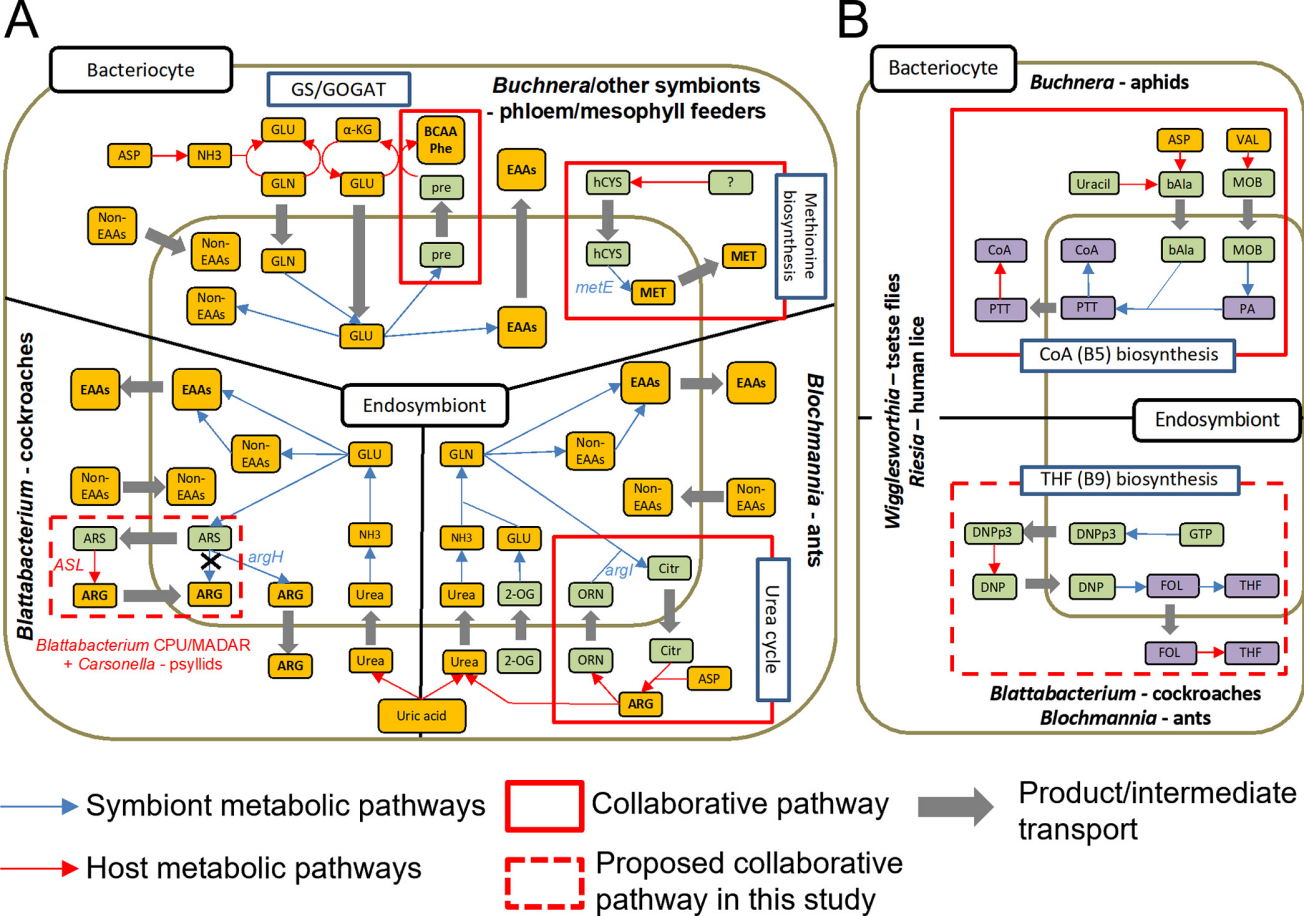
Schematic representations of metabolic interactions and distribution of collaborative pathways for amino acids biosynthesis among host-endosymbiont systems in insects. Narrow blue and red arrows indicate the metabolic pathways of the symbionts and hosts, respectively. Thick gray arrows indicate the transport of metabolites across membranes. Yellow, purple, and green boxes represent amino acids, vitamins/cofactors, and intermediate metabolites, respectively. (A) metabolic pathways for amino acid biosynthesis. (B) collaborative pathways for vitamin/cofactor biosynthesis. ARS, argininosuccinate; ORN, ornithine; Citr, citrulline; 2-OG, 2-Oxoglutarate; pre, precursors for BCAAs and phenylalanine; hCys, homocysteine; bAla, beta-alanine; PA, pantothenate; PTT, pantetheine; MOB, 3-methyl-2-oxobutanoate; DNPp3, 7,8-dihydroneopterin 3′-triphosphate; DNP, dihydroneopterin; FOL, folate; THF, tetrahydrofolate.

Inferred metabolic relationships between insects and their symbionts (11, 14, 22, 23, 28, 29, 34–68) are summarized in Table 1. Pathway predictions for other insect-symbiont pairs were performed using the same procedure used for *Blattabacterium*. Note that pathways present in secondary symbionts are omitted due to high variability in genome reduction levels. The data shown in Table 1 thus only include the relationship between the host and its primary symbiont. Overall, collaborative pathways are non-uniformly distributed across insect-symbiont pairs. While phloem-sap feeders tend to have many collaborations in EAA pathways, host-symbiont collaborative pathways in Blattodea and carpenter ants were only found in the arginine and folate biosynthetic pathways, and no collaborative pathways were found in the only xylem-sap feeder examined, Auchenorrhyncha-*Sulcia*. Both Blattodea and carpenter ants have predicted collaborative arginine biosynthetic pathways; however, the enzymes involved are different. Carpenter ants require ornithine carbamoyltransferase (ArgI: EC 2.1.3.3) from their *Blochmannia* endosymbionts to complement their arginine biosynthetic pathway (part of the urea cycle), while in Blattodea, CPUbr and MADAR require argininosuccinate lyase (ASL: EC 4.3.2.1) from their hosts to complement their *de novo* arginine biosynthetic pathway. In phloem-sap feeders, collaborative EAA, and in particular BCAA biosynthetic pathways, are especially common, regardless of the symbiont's taxonomic position. The commonness of collaborative BCAA biosynthetic pathways in these insects is likely due to the evolutionary stability of the interaction between host and symbiont. This stability is also found in standard biosynthetic pathways. For example, *Sulcia* strain OLIH, which has the genome among *Sulcia* strains used in this study, has lost genes for all essential amino acids and vitamins/cofactors except for those for BCAA biosynthesis (65). The *Blattabacterium* strains MADAR and CPUbr are in striking contrast to this pattern, as they have lost BCAA biosynthetic pathways together with several other EAA biosynthetic pathways. Another exception to this trend is found in partnerships between blood-sucking insects and their obligate endosymbionts, which have lost almost all EAA biosynthetic pathways while retaining almost all vitamin/cofactor pathways. For vitamins/cofactors, two collaborative biosynthetic pathways for pantothenate (B5) and folate (B9) biosynthesis were detected (Table 1). The former collaboration (B5) has been reported in aphids (69). In this study, Blattodea and carpenter ants (which feed on wood or are omnivorous) were predicted to have the latter (B9), and blood-feeders were predicted to have both (Fig. 5).

**TABLE 1:** Summary of the predicted metabolic interactions between primary endosymbionts and their insect hosts^*a*^

Symbiont strain (taxon)	Reference	Genome size (Kb)	Non-essential amino acids										Essential amino acids								Vitamins/Cofactors									
			Glu	Gln	Asp	Asn	Ser	Gly	Cys	Ala	Tyr	Pro	Phe	Trp	His	Thr	Lys	Met	Arg	BCAA	B1	B2	B3	B5	B6	B7	B9	K2	Hem	LA
Various																														
*Blattabacterium* Others (Fl)	22,23,34–36	640	O	H	O	H	O	H	O	O	O	H	B	B	B	B	B	B	B	B	-	B	-	-	B	-	C^g^	B	H	O
*Blattabacterium* Cp&Md (Fl)	28,29	600-590	O	H	O	H	O	H	O	O	O	H	B	-	B	-	B	-	C^g^	-	-	B	-	-	B	-	-	B	H	O
*Blochmannia* (Gp)	14,37–40	791-705	H	O/H	O	H	O/H	O	O	O	O	H	B	B	B	B	B	-	C^g^	B	-	B/-	-	-	B/-	-	C^g^	-	H	H
Phloem-sap																														
*Buchnera* (Gp)	5,41–45	655-422	O	H	H	H	H	O	O/H	O/H	H	H	C^g^	B/-	B	B	B	C^g^	B/C^g^	C^t^	-	B/-	-	C^t^/-	-	B/-	-	-	H	O/H
*Carsonella* (Gp)	46 – 48	166-159	O	H	H	H	H	O	H	H	H	H	C^t*^/-	-	B/-	B	B	-	B/C^t*^/-	B/C^g^	-	-	-	-	-	-	-	-	H	H
*Portiera* (Gp)^*a*^	49 – 52	359-280	H	H	H	H	H	H	H	O	H	H	C^t*^	B	C^t^^*b*^	B	C^t*^	C^t^	C^t*^	C^t^	-	-	-	-	-	-	-	-	H	O/H
*Tremblaya* (Bp)^*a*^	11,53,54	171-138	H	H	H	H	H	H	H	O	H	H	C^t^/-	B/-	B^*b*^	B	-	C^t^/-	C^g^/-	C^t^	-	-	-	-	-	-	-	-	H	H
*Uzinura* (Fl)^*a*^	55	263	O	H	O	H	H	H	H	O	H	H	B	B	B^*b*^	B	B	-	B	C^g^	-	-	-	-	-	-	-	-	H	O
*Walczuchella* (Fl)^*a*^	56	309	O	H	H	H	H	H	H	O	H	H	-	B	B^*b*^	B	B	-	B	C^g^	-	-	-	-	-	-	-	-	H	O
Xylem-sap																														
*Sulcia* (Fl)^*a*^	57 – 65	285-157	H	H	O/-	H	H	H	H	H	H	H	B/-	B/-	-	B/-	B/-	-	B/-	B	-	-	-	-	-	-	-	-	H	O
Blood																														
*Wigglesworthia* (Gp)	66,67	705	O	O	O	H	O	O	H	H	H	H	-	-	-	-	-	-	-	-	B	B	B	C^g^	B	B	C^g^/-	B	O	O
*Riesia* (Gp)	68	582	O	H	H	H	O	O	H	O	H	H	-	-	-	-	-	-	-	-	-	B	B^*b*^	C^g^	B	B	C^g^	-	H	O

a Predictions of metabolic interactions assuming that the insect hosts possess a standard insect gene set. B, bacteria-dependent; H, host-dependent; O, overlapping; C^g^, collaborative pathways predicted from genomic evidence; C^t^, collaborative pathways inferred from transcriptome evidence; C*, collaborative pathways with host genes of bacterial origins; -, absent from both the genomes of the host and primary symbiont; /, pathway completeness differs among strains in the group. Fl, *Flavobacteriales*; Gp, *Gammaproteobacteria*; Bp, *Betaproteobacteria*; Cp&Md, strain CPUbr and MADAR.

b Incomplete but assumed functional due to highly conserved remnant genes in the pathway.

## DISCUSSION

In this study, we analyzed the amino acid and vitamin/cofactor metabolic pathways of *Blattabacterium* endosymbionts from Blattodea and compared them to those of other *Flavobacteriales* genomes. We found that pathways for EAA biosynthesis are enriched in *Blattabacterium* genomes, while many non-EAA and vitamin/cofactor pathways have been lost. We further identified two types of gaps present in these pathways: (i) gaps shared by *Blattabacterium* and other *Flavobacteriales* strains, and (ii) gaps specific to *Blattabacterium*. We then analyzed the metabolic pathways of 3 species of Blattodea and compared them to those of *Blattabacterium* in order to examine potential metabolic collaborations in this symbiotic system. The product-level complementation (exchange of amino acids and vitamins between the host and its symbiont) shown in this study is consistent with results from previous studies, which performed genome-based flux balance analysis on *Blattabacterium* (70, 71). The pathways for several non-EAAs (Gln, Gly, Asn, and Pro) absent from *Blattabacterium* genomes were present in the transcriptome of *B. germanica* (72). While we found several examples of product-level complementation, we only found 2 candidate examples of within-pathway mutual dependence (defined as “collaborative pathways” in this study), in the folate biosynthetic pathway for all strains (except for CPUbr and MADAR), and in the arginine biosynthetic pathway for CPUbr and MADAR. We also compared metabolic pathways across 11 insect-symbiont systems comprised of 55 genomes belonging to various taxonomic groups and having diverse diets (Table 1, Table S3), and found that there is a clear pattern of putative collaboration related to the host diet. In addition to patterns related to diet, we also found that BCAA biosynthetic pathways have been retained by the endosymbionts of all sap-feeding insects examined here despite their significant levels of reductive genome evolution. In sharp contrast to this trend in sap-feeding insects, genome reduction in CPUbr and MADAR *Blattabacterium* involves the loss of BCAA pathways.

We found several unique characteristics in *Blattabacterium* metabolic pathways. Firstly, although urease is present in *Blattabacterium* and in the pathogenic strains of the genera *Weeksella*, *Ornithobacterium*, *Riemerella*, and *Chryseobacterium*, it is generally absent from the genomes of other *Flavobacteriales* genera. A previous study suggested that the presence of a urea transporter and urease accessory proteins is highly correlated with non-marine pathogenic *Flavobacteriales* ecotypes (73). Interestingly, our phylogenetic analysis of UreC protein suggests that *Blattabacterium* has acquired urease via horizontal gene transfer while almost all ureases from other *Flavobacteriales* strains are monophyletic (Fig. 2). The universal presence of this gene among all known *Blattabacterium* strains indicates it was present in their last common ancestor. The conversion of uric acid to usable nitrogen in the form of NH_3_ occurs via collaboration between the host (which performs the first steps [72]) and *Blattabacterium*. This process is an essential trigger for the biosynthesis of many nutrients (22, 23) and forms a key part of the symbiotic relationship between these partners. Other specific characteristics of the early stage of *Blattabacterium* genome evolution include losses of several non-EAA pathways and the retention of EAA pathways, which suggest that *Blattabacterium* has evolved with the strict requirement of supplying EAAs to their hosts.

Blattodea are known to contain a high level of cobalamin (B12) in their hindgut (74, 75). Since both host and *Blattabacterium* lack biosynthetic pathway of B12, the reported higher cobalamin concentration in the hindgut of Blattodea is likely due to the presence of B12-producing microbes in their hindgut, such as Shimwellia blattae (76). Unlike other animals, insects are not thought to require cobalamin as none of the cobalamin-dependent enzymes were detected from 19 publicly available genomes (77). We searched for the 3 cobalamin-dependent eukaryotic enzymes and could not detect them in the Blattodea genomes used in this study. We expect that the high level of B12 in the hindgut of Blattodea is not required by the host or its *Blattabacterium*.

Our study shows that the metabolic host-symbiont relationships in the Blattodea differ from those of other insects. With respect to collaborative pathways for amino acids biosynthesis, we propose 3 categories: (i) phloem-sap feeders, which commonly have collaborative BCAAs, phenylalanine, and methionine biosynthetic pathways; (ii) carpenter ants, which have a collaborative arginine biosynthetic pathway; (iii) and the 2 blattodeans *C. punctulatus* and *M. darwiniensis* and psyllids, which have another type of collaborative arginine biosynthesis pathway (Fig. 5A). Blood-feeders do not have any biosynthetic pathways for EAAs. Interestingly, while phloem-sap feeders have evolved similar patterns of collaboration, xylem-sap feeders do not appear to possess any collaborative pathways. With respect to vitamin/cofactor pathways, we propose 3 categories: (i) aphids, which have a collaborative pantothenate biosynthetic pathway; (ii) carpenter ants and Blattodea, which have collaborative folate biosynthetic pathways; (iii) blood-feeders, which have collaborative pathways for both pantothenate and folate (Fig. 5B).

A previous study suggested that host gene content can be a constraint for collaborative pathway evolution (10). In the case of BCAA biosynthesis, the branched-chain-amino-acid transaminase (BCAT: EC 2.6.1.42) is ubiquitous in insects, indicating that collaborative BCAA biosynthesis has the potential to evolve. Similarly, the 2 collaborative pathways in vitamin/cofactor biosynthesis we describe here are supported by the universal presence of the beta-alanine biosynthetic pathway and the alkaline phosphatase gene in insect genomes. The type of cell that gave rise to bacteriocytes is also thought to potentially constrain the evolution of collaborative pathways because the cells require adequate levels of gene expression to complement gaps in the symbiont’s pathways (10). The potential for forming collaborative pathways is thus expected to vary among different insect hosts, depending on gene expression levels in particular cells, even when the hosts possess the same set of genes. Previous studies have reported that within a host individual, different (primary and secondary) symbionts are harbored within morphologically distinct bacteriocytes (57, 78, 79), and the bacteriocytes have distinct gene expression profiles (80).

Although patterns of collaborative pathways across varying symbiotic systems are potentially explained by the “bacteriocyte cell type origin” hypothesis, our results suggest that collaborative pathways are driven by the host diet, and particularly the EAA and vitamin/cofactor profile required by the host. Previous studies have suggested that collaborative EAA biosynthesis is controlled by the host through control over amino donor supply (especially the supply of glutamate for BCAAs and phenylalanine) (3). The production rate of BCAAs and phenylalanine in aphid bacteriocytes was estimated to exceed 60% of total amino acid production (3). The glutamine synthetase/glutamine oxoglutarate aminotransferase (GS/GOGAT) cycle in the host cytoplasm is also thought to have a central role in efficient ammonia assimilation, which keeps toxic ammonia at low levels in bacteriocytes (8). Generally, phloem-sap is rich in asparagine and sugars, and thus symbiotic systems can obtain sufficient ammonia and ATP to activate the GS/GOGAT cycle. In contrast, xylem-sap is generally poorer in both carbon and nitrogen sources than phloem-sap, which might explain why Auchenorrhyncha-*Sulcia* systems lack collaborative pathways based on GS/GOGAT cycle. In this context, Blattodea do not need to use the GS/GOGAT cycle system because they use uric acid to store nitrogen wastes, and *Blattabacterium* utilizes host-provided urea as a nitrogen source (Fig. 5). Because Blattodea have various feeding habits, the EAA profile of their food source is less specific than plant sap. Therefore, control over the EAA production profile is likely to be less important in this system. The collaborative arginine biosynthetic pathway in the carpenter ant-*Blochmannia* system is believed to be linked to the use of arginine as a nitrogen storage compound in ants (14). This potentially explains the absence of the same type of collaborative arginine biosynthetic pathways found in carpenter ants and Blattodea. In termites, uric acid is transported into the gut via the malpighian tubules and degraded by gut microbiota (81). This may explain the absence of uricolytic ability in the genome of the lower termite *Z. nevadensis*, which has lost *Blattabacterium*.

Our study has revealed several general trends of metabolic pathway evolution within and across insects and their symbionts. We found genomic evidence that symbiont partnerships vary in their amino acid and vitamin/cofactor biosynthetic collaborations. Experimental evidence, such as differential gene expression analysis or enzymatic assays, is required to identify the precise mechanisms by which these collaborations occur. For example, in the case of gaps unique to one or several strains of symbionts, complementation by unknown host enzymes or the emergence of new multifunctional enzymes should be considered as potential replacements (69). Our analyses revealed several gaps specific to *Blattabacterium* and several gaps common across *Flavobacteriales*. However, since our approach focused on interpreting gaps rather than reconstructing whole system metabolic networks, it is unclear how the metabolic collaborations affect the total metabolic flux within the symbiotic system. Although multi-organism scale modeling methods are needed to answer such questions, these modeling methods require experimental evidence, such as metabolite analyses, chemically-defined diets, or differential gene expression data, to make accurate predictions. Future studies are needed to determine the precise mechanisms and adaptive significance of pathway complementation in insect-symbiont systems and to provide a clear picture of the evolution of their genomes.

## MATERIALS AND METHODS

### Genome and transcriptome data of hosts.

We used the genomes of 2 species of Blattodea, *Periplaneta americana* (82) and *Zootermopsis nevadensis* (83), and the transcriptome of a cockroach, *Cryptocercus punctulatus* (84), to infer the common metabolic pathways of Blattodean hosts. Although *Z. nevadensis* does not harbor *Blattabacterium*, we assumed that the metabolic pathways shared among these three distantly related species are common across Blattodean hosts. We did not use the published genome of the cockroach *Blattella germanica* (85) because its completeness, evaluated with BUSCO ver. 4 (86), was markedly lower than that of the 2 genomes and the transcriptome publicly available (Table S1). Because gene prediction was not available for the genome of *P. americana*, we conducted gene prediction using a transcriptome data set of *P. americana* generated by another study (87). Details concerning gene prediction and transcriptome assembly are available in the supplementary methods.

### Genomes of *Blattabacterium* and related *Flavobacteriales* bacteria.

The genomes of 7 strains of *Blattabacterium* were used for metabolic reconstruction. We also reconstructed the metabolic pathways of 42 genomes of other *Flavobacteriales* bacteria that are not obligate mutualists and one genome from the order *Bacteroidales*. All bacterial genomes used for the metabolic reconstruction are listed in Table S2. Phylogenetic positions of *Flavobacteriales* bacterial genomes are shown in Fig. S1.

### Phylogenetic tree inference.

Orthologous genes present in all studied genomes of *Flavobacteriales* (Table S2) were determined using Proteinortho5 (88) with >0.9 cluster quality threshold. Thirty-one representative marker genes for bacteria were extracted from orthologous sets using AMPHORA2 (89). Extracted sequences were aligned using MAFFT ver. 7 (90) in “linsi” mode, and ambiguously aligned sites were removed with Gblocks (91) using default parameters. Trimmed amino acid alignments were checked for gene conversion using Genconv under default parameters and concatenated with FASconCAT-G (92). All sequences of the concatenated alignment were re-coded into a Dayhoff6 matrix to reduce potential biases due to base composition heterogeneity between bacterial species (93). A maximum likelihood phylogenetic tree was calculated using RAxML ver. 8 (94) with the GTR-MULTIGAMMA substitution model. Branch support was estimated using 100 bootstrap replicates. For the UreC protein, all available bacterial and archaeal sequences were obtained from OrthoDB v10 (95), and the sequences were filtered with respect to length (sequences with <550 and >650 amino acids were removed). The remaining sequences, except for *Flavobacteriales*, were further filtered using the VSEARCH clustering method (96) with a 75% identity threshold. Alignments were prepared as described above. A maximum likelihood phylogenetic tree was reconstructed using RAxML ver. 8 with the PROTGAMMALG amino acid substitution model.

### Metabolic reconstruction.

We carried out metabolic reconstructions for the hosts, *Blattabacterium*, and several strains of free-living bacteria belonging to *Flavobacteriales*. Because annotations based solely on sequence homology occasionally contain inaccurate assignments (97), enzyme-coding genes were identified and re-curated using a “profile homology” information approach. First, 2 profile-based annotation programs, InterProScan (98), and SPARCLE (99), were run against all CDSs of the genome. Each CDS with an E-value <1E-10 was given an enzyme commission (EC) number using EC2GO mapping (100). We used EC assignments with four and 3 digits (i.e., EC X.X.X.X or EC X.X.X.-) to reduce ambiguous EC assignments. This domain-based method is known to have high accuracy (100); nonetheless, some enzyme families and subfamilies were not appropriately annotated using this method (101). Therefore, the sequences with no EC assignment were annotated using RPSBLAST searches against the COG database (102). The final set of EC numbers and COG IDs were mapped onto MetaCyc pathways (103), and the direction of each reaction was inferred, when possible, using information from the BRENDA database (104).

We investigated whether gaps or missing enzymes in *Blattabacterium* metabolic pathways were also found in related bacteria. Gaps conserved across related bacterial lineages imply the presence of alternative/unknown enzymes complementing the pathways. Phylogenetic comparative methods have previously been used to identify novel genes completing gaps in metabolic pathways (105–107). In this study, we focused on determining whether pathways with gaps are complemented. We did not attempt to identify the enzymes filling the gaps. We investigated all biosynthetic pathways having up to 2 gaps. We assumed that gaps are complemented by alternative enzymes if they are shared by over 70% of other *Flavobacteriales* genomes. We refer to these gaps as “common gaps,” which all share three characteristics: (i) evolutionary stability; (ii) lineage-specificity, implying they are reactions which are complemented by alternative/unknown enzymes (25, 108); and (iii) representative of the ecological diversity of Flavobacteriales bacteria (109). The former 2 characteristics strongly support that the pathways with common gaps are functional, and the third characteristic supports that the dependence on the same metabolic intermediates is unlikely. Gaps were considered specific to *Blattabacterium* when shared by the 7 *Blattabacterium* genomes analyzed here but present in less than 10% of other *Flavobacteriales*. We refer to these gaps as “specific gaps”. Specific gaps were assumed to be complemented by the host if enzymes catalyzing the same reaction were found in the host genome. In case no such enzymes were found in the host genome, we assume the presence of alternative enzymes specifically found in *Blattabacterium*.
