# Supplementary material for: Coevolution of Metabolic Pathways in Blattodea and Their Blattabacterium Endosymbionts, and Comparisons with Other Insect-Bacteria Symbioses
Source: Microbiol Spectr. 2022 Sep 12;10(5):e02779-22. doi: 10.1128/spectrum.02779-22 (PMC9603385; doi:10.1128/spectrum.02779-22)
Supplement: Supplemental file 1 — Tables S1 and S2, Fig. S1, and supplemental methods. Download spectrum.02779-22-s0001.pdf, PDF file, 0.2 MB [file spectrum.02779-22-s0001.pdf]

Coevolution of metabolic pathways in Blattodea and their *Blattabacterium* endosymbionts, and comparisons with other insect-bacteria symbioses

Yukihiro Kinjo[1,2,3*], Thomas Bourguignon[1,4], Yuichi Hongoh[2,3], Nathan Lo[5], Gaku Tokuda[6], and Moriya Ohkuma[2]

Supplementary Materials

Table S1 Genome and transcriptome data used for metabolic pathway inferences in this study.

	Blattodea species			
	B. germanica	*P. americana*	*C. punctulatus*	*Z. nevadensis*
Data Type	Genome	Genome	Transcriptome	Genome
Proteins/Transcripts	27,928	52,813	35,718	15,904
Proteome completeness	72.0*	93.0*	88.1*	97.8
Reference	Harrison et al. 2018	Li et al. 2018	Hayashi et al. 2017	Terrapon et al. 2014
Data acssession	GCA_003018175.1	GCA_002939525.1	DRA001254	GCA_000696155.1

* Proteins with best hit to non-Arthropoda organisms were filtered out.

Table S2 Bacterial genomes used for species tree inference and metabolic pathway reconstructions in this study.

Strain (host species)	Refseq accession	Genome size (Mbp)	Gene
Blattabacterium sp. str. BNCIN (*Nauphoeta cinerea*)	CP005488.1	0.63	621
Blattabacterium sp. str. BPLAN (*Periplaneta americana*)	CP001429.2	0.64	633
Blattabacterium sp. str. Bge (*Blattella germanica*)	CP001487.1	0.64	624
Blattabacterium sp. str. MADAR (*Mastotermes darwiniensis*)	CP003000.1	0.59	589
Blattabacterium sp. str. CPUbr (*Cryptocercus punctulatus*)	CP029816.1	0.61	587
Blattabacterium sp. str. BGIGA (*Blaberus giganteus*)	CP003535.1	0.63	615
Blattabacterium sp. str. BOR (*Blatta orientalis*)	CP003605.1	0.64	625
Blattabacterium sp. str. BPAA (*Panesthia angustipennis spadica*)	AP012548.1	0.63	622
Candidatus Uzinura diaspidicola str. ASNER (*Aphytis melinus*)	CP003263.1	0.26	272
Candidatus Sulcia muelleri str. GWSS (*Homalodisca vitripennis*)	CP000770.2	0.25	264
Candidatus Sulcia muelleri str. CARI (*Clastoptera arizonana*)	CP002163.1	0.28	288
Candidatus Walczuchella monophlebidarum (*Llaveia axin axin*)	CP006873.1	0.31	313
Capnocytophaga ochracea DSM 7271	CP001632.1	2.61	2216
Cellulophaga lytica DSM 7489	CP002534.1	3.77	3311
Chryseobacterium sp. StRB126	AP014624.1	5.50	4951
Croceibacter atlanticus HTCC2559	CP002046.1	2.95	2693
Dokdonia sp. MED134	CP009301.1	3.30	2919
Flavobacteriaceae bacterium 3519-10	CP001673.1	2.77	2543
Flavobacterium indicum DSM 17447	HE774682.1	2.99	2769
Flavobacterium johnsoniae UW101	CP000685.1	6.10	5219
Flavobacterium psychrophilum	CP012586.1	4.14	3634
Formosa agariphila KMM 3901	HG315671.1	4.23	3568
Gramella forsetii KT0803	CU207366.1	3.80	3429
Maribacter sp. HTCC2170	CP002157.1	3.87	3386
Myroides profundi	CP010817.1	4.06	3608

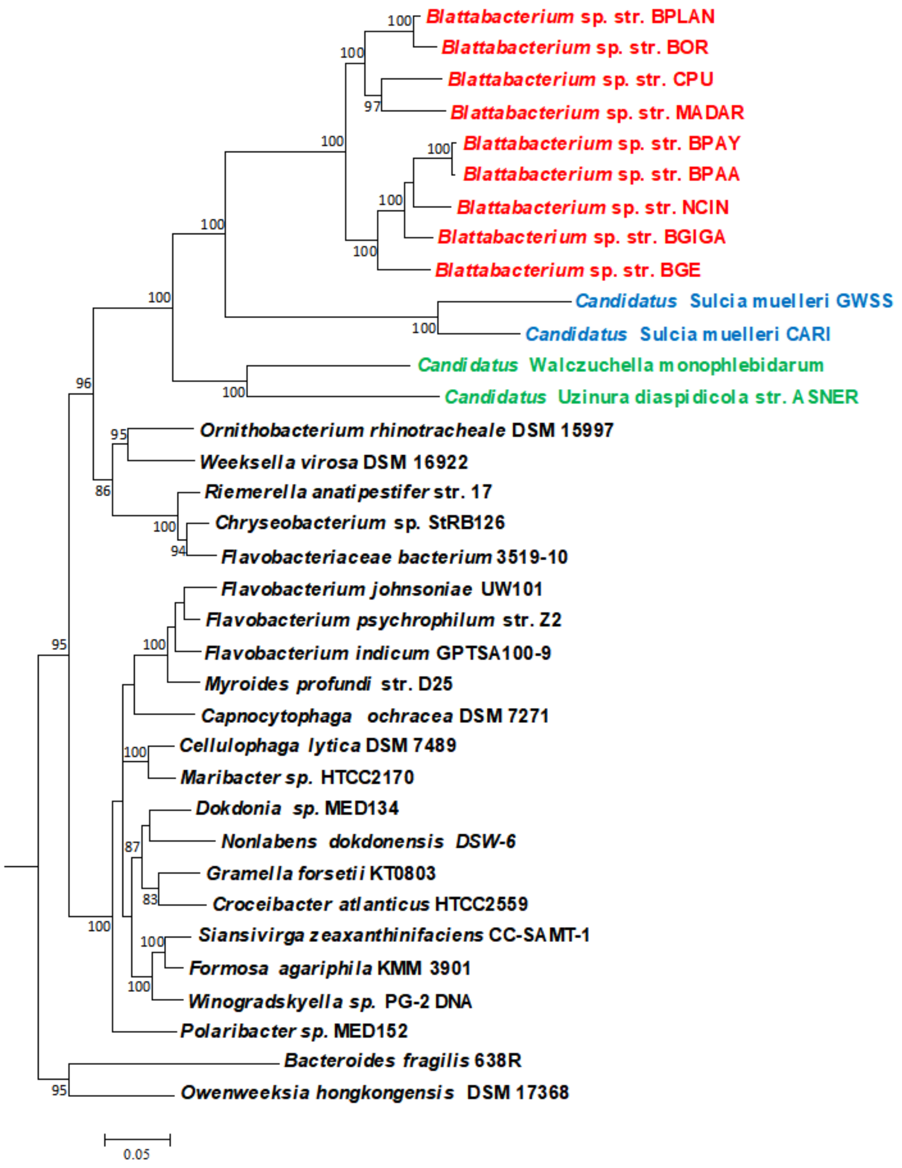

Fig. S1. Maximum likelihood phylogenetic tree of *Flavobacteriales* reconstructed using RAxML with GTR-MULTIGAMMA model.

The tree was inferred from 22 conserved single-copy orthologous genes using Dayhoff6-recoded amino acid matrix. Branch labels indicate bootstrap support values greater than 80. Strain names with colored characters are obligate endosymbionts of insects (red; Blattodea, blue; Auchenorrhyncha, green; Coccoidea).

Supplementary methods

Transcriptome assembly and ORF prediction

Illumina HiSeq 2000 reads from *P. americana* and *C. punctulatus* transcriptome sequencing projects were downloaded from NCBI Sequence Read Archive (DRA001254 and DRA004598, respectively). Adapters and low-quality regions (<30 phred score) were removed from the reads using FaQCs (1). Reads with low average quality score (<30) were also removed with FaQCs. The remaining high-quality reads were assembled with Trinity ver. 2.2 (2) using the following settings: "min_kmer_cov 2" and "normalize_reads". Protein-coding open reading frames (ORFs) of the assembled transcriptomes were predicted with TransDecorder (3) using the homology search option (blastp and Pfam search against Swiss-prot and Pfam-A database, respectively). To remove potential sequence contaminations from other organisms (e.g., bacteria and fungi), we extracted ORFs assigned to Arthropoda proteins by a megablast search implemented in BLAST+ software (4) against NCBI non-redundant (NR) database as a reference. We used those extracted ORFs for enzyme prediction and metabolic reconstruction analyses. Completeness of the proteomes translated from the ORFs were evaluated with BUSCO ver.4 (5) based on an Arthropoda conserved gene set (arthropoda_odb10).

Gene prediction on **P. americana** *genome*

Genome sequence of *P. americana* was obtained from GenBank (accession: GCA_002939525.1). To remove potential sequence contaminants (e.g. such as bacterial and/or fungal sequences), all contigs were submitted to BLASTX homology search against NCBI NR database by using DIAMOND ver. 2.0.4 (6). The contigs which have best hits only on the sequences other than Arthropoda were assumed to

be contaminant contigs and then removed from downstream analyses. Candidates of repetitive regions in the filtered genome sequence were identified by RepeatModeler ver. 2.0.2 (7), and were then soft-masked by RepeatMasker ver. 4.1.2 (8). Gene prediction on the genome sequence was done by using BRAKER pipeline ver. 2.1.4 (9) with "etp mode" to obtain better completeness of gene prediction. RNAseq reads from the *P. americana* transcriptome (DRA001254) and a proteome data from OrthoDB v10 (10) Arthropoda gene set (odb10_arthropoda_fasta) were used as reference dataset. To recover the genes which were not predicted by the pipeline, the translated proteome from assembled *P. americana* transcripts were compared with the proteome predicted by the pipeline, and the proteins which were present in the transcriptome but not in the predicted proteome were manually curated.

References

1. Lo C-C, Chain PSG. 2014. Rapid evaluation and quality control of next generation sequencing data with FaQCs. BMC Bioinformatics 15.
2. Grabherr MG, Haas BJ, Yassour M, Levin JZ, Thompson DA, Amit I, Adiconis X, Fan L, Raychowdhury R, Zeng Q, Chen Z, Mauceli E, Hacohen N, Gnirke A, Rhind N, di Palma F, Birren BW, Nusbaum C, Lindblad-Toh K, Friedman N, Regev A. 2011. Full-length transcriptome assembly from RNA-Seq data without a reference genome. 7. Nat Biotechnol 29:644–652.
3. Haas BJ, Papanicolaou A, Yassour M, Grabherr M, Blood PD, Bowden J, Couger MB, Eccles D, Li B, Lieber M, MacManes MD, Ott M, Orvis J, Pochet N, Strozzi F, Weeks N, Westerman R, William T, Dewey CN, Henschel R, LeDuc RD, Friedman N, Regev A. 2013. De novo transcript sequence reconstruction from RNA-Seq: reference generation and analysis with Trinity. Nat Protoc 8.
4. Camacho C, Coulouris G, Avagyan V, Ma N, Papadopoulos J, Bealer K, Madden TL. 2009.

BLAST+: architecture and applications. BMC Bioinformatics 10:421.

5. Seppey M, Manni M, Zdobnov EM. 2019. BUSCO: Assessing Genome Assembly and Annotation Completeness, p. 227–245. *In* Kollmar, M (ed.), Gene Prediction: Methods and Protocols. Springer, New York, NY.

6. Buchfink B, Xie C, Huson DH. 2015. Fast and sensitive protein alignment using DIAMOND. Nat Methods 12:59–60.

7. Flynn JM, Hubley R, Goubert C, Rosen J, Clark AG, Feschotte C, Smit AF. 2020. RepeatModeler2 for automated genomic discovery of transposable element families. Proc Natl Acad Sci 117:9451–9457.

8. Saha S, Bridges S, Magbanua ZV, Peterson DG. 2008. Empirical comparison of ab initio repeat finding programs. Nucleic Acids Res 36:2284–2294.

9. Brůna T, Hoff KJ, Lomsadze A, Stanke M, Borodovsky M. 2021. BRAKER2: automatic eukaryotic genome annotation with GeneMark-EP+ and AUGUSTUS supported by a protein database. NAR Genomics Bioinforma 3.

10. Kriventseva EV, Kuznetsov D, Tegenfeldt F, Manni M, Dias R, Simão FA, Zdobnov EM. 2019. OrthoDB v10: sampling the diversity of animal, plant, fungal, protist, bacterial and viral genomes for evolutionary and functional annotations of orthologs. Nucleic Acids Res 47:D807– D811.
